# What community-based public health approaches in West Africa for COVID-19 epidemic? A reflection based on the African socio-cultural context

**DOI:** 10.11604/pamj.supp.2020.35.2.24415

**Published:** 2020-06-25

**Authors:** Ndèye Marème Sougou, Jean-baptiste Diouf, Mamadou Makhtar Mbacké Leye, Oumar Bassoum, Adama Faye, Ibrahima Seck

**Affiliations:** 1 Department of Preventive Medicine and Public Health, Cheikh Anta Diop University of Dakar, Senegal; 2 Institute of Health and Development, Cheikh Anta Diop University of Dakar, Senegal; 3 Hospital Roi Baudouin, Dakar, Senegal

**Keywords:** COVID-19, community-based interventions, West-Africa

## Abstract

The social and cultural dimensions of health influence the course of disease and condition the success of health interventions. In Africa, previous epidemics such as Ebola have shown the importance of contextualizing health interventions. This literature review contributes to the reflection on the analysis of community-based interventions in the context of the particularities of West Africa in the fight against the pandemic in COVID-19.

## Essay

The potential contribution of socio-anthropology is based on the empirical comparison of particular societies. It thus makes it possible to address the social and cultural dimensions of health, ill health and medicine [1]. Previous epidemics on the African continent (Ebola), had highlighted the hostility of populations to unpopular measures, not socially accepted because they were out of step with local cultural codes [2]. The Ebola epidemic in West Africa has shown the usefulness of social sciences in dealing with epidemics. Experience of the contribution of social sciences has been gained in field action [3]. This reflection within the framework of this pandemic concerning COVID-19 will focus in particular on the analysis of community interventions with regard to the contextual particularities of West Africa. Questions will be raised about the place to be given to the fight against VIDOC at Community level. As a reminder, the community level of health seeks to reduce social inequalities in health by employing an empowerment process based on community involvement [4].

WHO recommendations [5] (World Health Organization) regarding the control of COVID-19 in community settings are as follows: (1) Avoid groups of people and closed and crowded spaces; (2) maintain a physical distance of at least 1 m from others people, especially those with respiratory problems symptoms (e.g., coughing, sneezing); (3) Wash hands frequently, using an alcohol-based product-Rub hands if they are not visibly soiled or if they are washed with soap and water when hands are visibly soiled; (4) Cover your nose and mouth with a folded elbow or paper tissue in case of cough or sneeze, remove immediately after use, and wash your hands; (5) refrain from touching your mouth, nose and eyes. According to these recommendations, in some countries, masks are worn in accordance with local customs or as advised by national authorities within the framework of COVID-19. In these situations, best practice should be followed with regard to the wearing, removal and disposal of masks, as well as hand hygiene after their removal.

The questions that can be asked in relation to the various recommendations are:

In relation to the rules of social distancing: 1) Is it socially accepted, and physically possible, to protect oneself by following strict rules of social distancing or is it only a theoretical fiction in the context of West African families and sociability? 2) What community-based solutions can be recommended in these contexts of the COVID-19 epidemic in West Africa? 3) Social distancing measures have always been part of the measures taken in cases where the virus was highly infectious and therapeutic management measures were not yet available (vaccine and antiretrovirals). This was the case with the Asian flu of 1957-58, which had a high potential to infect (≈50% of people infected). Closing schools and keeping children and teenagers at home reduced the attack rate by more than 90% [6]. It should be noted that social distancing measures include bans on large gatherings, including the cancellation of major events, the closure of schools and the reduction of close contact between people through behavioral change [7]. These measures have proven to be effective as a means of combating epidemics [8].

When talking about African societies and their values, reference is always made to the pre-eminence of the ideals carried by families, including solidarity among members of an extended family group [9]. These values have indeed structured and are still structuring African societies, all the more so as countries are finding it very difficult to bring out new constituent values. Today, these countries are still changing societies where traditionalism and modernism coexist. In Senegal, for example, joking cousinhood, a symbolic representation of traditional society, continues to be an instrument for strengthening social ties and thus reducing social distancing. Identity or status negotiations do not exhaust uses. In this way, customs are reproduced and transformed also and above all because of their practical uses, which are very diverse, but whose common objective is the establishment of a privileged relationship between men [10]. In addition, according to UN-HABITAT, West Africa is the seat of uncontrolled urbanization where large numbers of people are crowded into informal, irregular and unplanned neighborhoods [11]. In 2017, there were 472 million urban dwellers in sub-Saharan Africa, most of whom lived in informal or irregular settlements [12]. The expansion of COVID-19 could therefore benefit from this context of uncontrolled urbanization in West Africa.

It emerges from this analysis that respecting social distancing as measures to combat the COVID-19 pandemic is difficult to respect taking account of social values of west African societies and designing of West African cities. However, there are levers that can be used to promote adherence to distancing in West African countries. Indeed, in most of these countries, there is a community organization based on the leadership of certain categories of population [13]. We will take the example of the traditional chieftaincy in Niger, which established itself as an essential community structure in the administration of community interventions or community relays in Senegal as a pioneer of a health intervention (seasonal intermittent preventive treatment of malaria) [14,15]. It is thus possible to identify the community structures that can influence the promotion of measures to fight COVID-19 in West Africa considering the specificities of each country.

**Could we therefore refer to the wearing of masks and/or hand hygiene as a key preventative measure?** studies have shown that face masks and hand hygiene can prevent infection in community settings, provided they are used and adhered to early [16,17]. We did not find any economic analyses in the literature concerning the cost of using face masks in the event of an epidemic. However, a study conducted in the United States during the H1N1 pandemic in 2009 showed that the use of masks could significantly reduce economic losses due to a pandemic [18]. Globally, the use of reusable cloth masks is widespread, particularly in Asia, which is an important region for emerging infections, but there is a lack of clinical research to inform their use and most policies do not provide guidance on their use [16]. The social interest of these masks remains the minimization of costs for the user. This interest is all the higher in developing countries in West Africa where the majority of the population lives below the poverty lines.

With regard to the hand hygiene measure, studies have shown that handwashing practices are poor in some West African countries [19]. In some West African countries, there are certain beliefs that would impact on hand hygiene [20]. This is the case in Senegal, where it has been determined that the use of soap during ritual cleansing before going to pray at the mosque is supposed to remove purity from spiritual cleansing because blessed water is not compatible with soap [21]. In addition, this situation of poor handwashing practices could be explained by the fact that in some communities in these countries there is a shortage of households with improved sanitation facilities on the one hand and a lack of behavioral support for better hygiene on the other [22]. During the Ebola outbreak in some of the West African countries, improved handwashing practices were found as a result of health promotion interventions. This change in handwashing practices was associated with exposure to health promotion information and communication channels. These included the mass media and religious and community leaders. However, the main reason for the change in handwashing practices was related to the perception of the disease by the Ebola virus communities, as Ebola is considered deadly [23]. Studies have concluded that extensive public education is needed to reduce the risk of infection associated with poor handwashing and special attention should be paid to the dissemination of information in future epidemics in Africa [19,24].

As a result of these reflections supported by the literature, recommendations for control at the community level in West Africa should revolve around the following points: The use of face masks by all members of the community at all times; Further research focusing on evaluating the effectiveness of fabric masks and studying current practices such as mask reuse; Hand hygiene promotion with soap and water; Health promotion using the mass media with a focus on a better understanding of the disease at COVID-19 and not just barrier gestures; The involvement of networks and community leaders in disease control strategies would promote protective measures such as social distancing.

## Competing interests

The authors declare no competing interests.
